# Association of clinical, imaging and laboratory parameters with adverse effects of glucocorticoid therapy in patients with giant cell arteritis

**DOI:** 10.3389/fmed.2024.1382946

**Published:** 2024-05-22

**Authors:** Leyla Schweiger, Franz Hafner, Andreas Meinitzer, Marianne Brodmann, Christian Dejaco, Philipp Jud

**Affiliations:** ^1^Division of Angiology, Department of Internal Medicine, Medical University of Graz, Graz, Austria; ^2^Institute of Medical and Chemical Laboratory Diagnostics, Medical University Graz, Graz, Austria; ^3^Division of Rheumatology, Department of Internal Medicine, Medical University of Graz, Graz, Austria; ^4^Department of Rheumatology, Hospital of Brunico (SABES-ASDAA), Brunico, Italy

**Keywords:** giant cell arteritis, adverse effects, glucocorticoids, inflammation, endothel dysfunction

## Abstract

**Background:**

Giant cell arteritis (GCA) is characterized by inflammation of large and medium vessels. First-line therapy for the treatment of GCA are glucocorticoids, which are effective while potential adverse effects should be considered, especially during long-term use. The aim was to investigate the incidence of glucocorticoids’ adverse effects and potential predictors for them.

**Materials and methods:**

138 GCA patients were retrospectively evaluated for newly developed glucocorticoid adverse effects in 2020. Potential predictors, defined as initial glucocorticoid pulse therapy, relapse of GCA and concomitant polymyalgia rheumatica as well as parameters of inflammation and endothelial dysfunction, including pulse-wave velocity and intima-media-thickness, were measured in 2012.

**Results:**

Potential new glucocorticoid adverse effects per patient was 1 (25th-75th 0–3) of which chronic kidney disease progression (29%), bone fractures (23.2%), cataracts (18.1%), dementia, and arterial hypertension (each at 12.3%) were most commonly recorded. Significant associations were found between occurrence of any relapse and new diabetes mellitus and between initial glucocorticoid pulse therapy and new dementia (all with *p* < 0.05). In multivariate regression analysis, any relapse was a predictor for developing diabetes mellitus (OR 9.23 [95% CI 1.33–64.05], *p* = 0.025). However, no correlations were observed between endothelial dysfunction or inflammatory parameters and development of new glucocorticoid adverse effects.

**Conclusion:**

GCA relapses may be associated for development of diabetes mellitus potentially by increasing glucocorticoid doses. Parameters of inflammation and endothelial dysfunction are not suited predictors for glucocorticoid adverse effects.

## Introduction

Giant cell arteritis (GCA) is classified as a large vessel vasculitis and is the most prevalent form of systemic vasculitis in adults with an annual incidence rate of 15–25 cases per 100,000 individuals (1). This condition primarily affects individuals over the age of 50 and it tends to be more common among women than men (2, 3). GCA is characterized by an inflammatory process that primarily affects large and medium-sized arteries, including the aorta and extracranial branches of the carotid arteries. This inflammatory process may lead to substantial damage, potentially resulting in complications like stenosis, occlusions, and even aneurysms in the affected arteries (4–6). The clinical presentation of GCA encompasses a range of symptoms, such as unilateral or bilateral temporal headaches, myalgia, jaw claudication, fatigue, and acute visual impairment (7).

In line with the recent recommendations from the European Alliance of Associations for Rheumatology (EULAR), glucocorticoids are the first-line therapy for GCA, particularly involving high doses when ocular complications are present (8). Although glucocorticoids are the most used therapy for GCA, this form of treatment is associated with a multitude of potential adverse effects exhibiting a dose-dependent pattern. Prolonged usage of glucocorticoids is associated with typical adverse effects, including osteoporosis, gastritis, arterial hypertension, and the onset of diabetes mellitus (9–11). Additionally, the risk for venous thromboembolism (VTE) is 3.5-fold higher during treatment with glucocorticoids and vascular dementia was also more likely to be diagnosed in those patients that had ever used long-lasting glucocorticoid treatment of more than two years (12, 13). Furthermore, it has been reported that adverse effects of glucocorticoid therapy may occur in up to 86% of GCA patients (10). Due to those adverse effects, glucocorticoid tapering need to done during an inactive phase of GCA, while relapse of GCA may occur during glucocorticoid tapering and relapse rates may be higher upon withdrawal of glucocorticoids (10, 14, 15). Therefore, determining the most effective treatment strategy to prevent relapse and minimize glucocorticoid adverse effects in GCA is challenging. Moreover, potential risk factors predicting glucocorticoid adverse effects are rarely described, especially GCA-specific parameters have been scarcely evaluated.

The aim of this study was to investigate the incidence of adverse effects caused by glucocorticoid therapy and find potential predictors for these effects in patients with GCA.

## Materials and methods

### Study design and patient cohort

This is a sub-study of a previously published study investigating cardiovascular diseases in patients with GCA (16). In brief, patients with a diagnosed GCA between 1993 and 2010 were identified by electronic search and invited to participate that study in 2012. At study inclusion between January and December 2012, blood sampling for parameters of endothelial dysfunction and inflammation, ultrasound measuring intima-media-thickness (IMT), and pulse-wave analysis measuring arterial stiffness were performed. All measurements were performed in a phase of inactive GCA and no subject had a disease relapse within a period of at least six months prior to study inclusion. After study inclusion, patients were followed-up by clinical routine. Charts review was performed in 2020 retrieving retrospectively patients’ demographics and clinical parameters up to study inclusion and recording retrospectively potential newly developed glucocorticoid adverse effects and relapse of GCA after study inclusion.

Patients with GCA were diagnosed clinically by the treating angiologic or rheumatologic physician based on clinical parameters, laboratory data, imaging and/or biopsy. All patients had been diagnosed with GCA of at least two years prior study inclusion. The modified criteria from the American College of Rheumatology (ACR) proposed by Dejaco et al. (17) were fulfilled retrospectively in all GCA subjects. Exclusion criteria for GCA patients were active cancer, infections, or other types of vasculitis.

### Laboratory parameters

Fasting blood samples for evaluation of inflammatory parameters, including C-reactive protein (CRP), erythrocyte sedimentation rate (ESR), fibrinogen, and white blood cells including lymphocyte subsets, were obtained from each patient at study inclusion in 2012. Additionally, CRP, ESR and fibrinogen from the time of GCA onset have been collected retrospectively. Peripheral blood mononuclear cells were isolated by Histopaque density gradient centrifugation and total cell number was determined by a Beckmann Coulter for measurement of lymphocytes subsets. Surface staining was performed according to routine protocols using appropriate combinations of antibodies for detection of CD3, CD4, CD8, CD28, CD45RA, CD45RO and appropriate isotype controls. Stained cells were measured using a fluorescence-activated cell sorter Canto II (Becton Dickinson), and data analysis was conducted with DIVA software and FlowJo. For the measurement of asymmetric dimethylarginine (ADMA) and symmetric dimethylarginine (SDMA) by high-performance liquid chromatography as described by Meinitzer et al. (18), one tube of whole blood was collected at study inclusion and subsequently centrifuged at 4,000 g for 10 min at 15°C temperature within 1 h after blood sampling obtainment. The supernatant was collected and divided into aliquots of 1 mL, which were stored at −80°C until final analysis.

### Imaging parameters

Details about measurements of IMT and arterial stiffness have been described previously (19). In brief, IMT of both common carotid, both subclavian and both common femoral arteries was measured by ultrasound using a linear transducer with 8–13 MHz (Siemens ACUSON S2000^™^, Siemens Healthcare Corp., Henkelstr., Erlangen, Germany) manually on magnified frozen longitudinal images and present carotid IMT of ≥0.9 mm in any common carotid artery was defined as abnormal (20, 21). Subsequently, carotid-femoral pulse-wave velocity (PWV) and augmentation index (Aix) were measured and calculated by automated analysis via photo-plethysmographic device Vascular Explorer® (enverdis Ltd., Fürstenwall, Düsseldorf, Germany) using software version 1.0 defining PWV >10 m/s as pathologic (20).

### Charts review of glucocorticoid adverse effects and clinical parameters

Charts review from all GCA subjects was performed between July and December 2020 via a fully electronic patient information system, called Medical Documentation and Communication network of Styria (MEDOCS), which is installed in the province of Styria, Austria, to provide electronic health data from all public Styrian hospitals and hospital alliances (22). Patient’s demographics, clinical parameters, defined as initial glucocorticoid pulse therapy, relapse and concomitant polymyalgia rheumatica (PMR), and potential prevalent glucocorticoid adverse effects prior to study inclusion in 2012 were recorded. Additionally, potential newly developed glucocorticoid adverse effects and relapse during follow-up were recorded. Potential adverse effects of systemic glucocorticoid therapy were defined as arterial hypertension, diabetes mellitus, obesity, hyperlipidemia, including hypercholesterolemia and hypertriglyceridemia, chronic kidney disease (CKD), osteoporosis, bone fracture, cataract, glaucoma, hepatic steatosis and cirrhosis, VTE, depression, dementia, gastritis, peptic ulcer, esophagitis, and pancreatitis (22, 23). Definition of the respective glucocorticoid adverse effect was made by adoption of the respective diagnosis from another hospital and/or by respective investigation, like measurement of the estimated glomerular filtration rate with subdivision into CKD 1–5 according to the recent KDOQI classification for CKD, X-ray densitometry for osteoporosis or abdominal sonography for hepatic steatosis. Relapse was defined as major or minor relapse according to the EULAR recommendations for the management of large vessel vasculitis (8). The end of the follow-up period was patient’s last documented medical report in MEDOCS.

### Statistics

Normally distributed parameters were expressed as means ± standard deviation (SD), non-normally distributed parameters as median with interquartile range and categorical parameters as frequency and percentages. Normality of distribution was examined by the Kolmogorov–Smirnov test and visual inspection. Assessment for the association between glucocorticoid adverse effects and clinical parameters of GCA was done by chi-square test and by simple as well as multiple logistic regression analyses. Multiple regression analysis was adjusted for important confounding variables, including age, sex, active smoking, arterial hypertension, diabetes mellitus and obesity. Pearson’s and Spearman‘s correlation coefficients were utilized for normally and for non-normally distributed variables, respectively. Given an exploratory study character no adjustment for multiple testing was applied. Statistical significance was assumed for *p* values <0.05. Statistical analyses were executed via SPSS version 27.0.

### Ethic approval and informed consent

This study was approved by the local ethics committee of the Medical university of Graz (EK Nr. 32–469 ex 19/20) and was conducted in accordance with the recent Helsinki Declaration. All patients provided written informed consent at study inclusion.

## Results

138 patients with GCA (106 female, 76.8%) with a mean age (± SD) of 74.5 ± 7.7 years were included in this study. Most common potential previously known glucocorticoid adverse effects at study inclusion were CKD (93.5%) followed by arterial hypertension (70.3%) and hyperlipidemia (66.7%). Further potential previous glucocorticoid adverse effects, concomitant medications at baseline and selected laboratory parameters at GCA onset are shown in Table 1.

**Table 1:** Patients’ characteristics and retrospectively collected potential glucocorticoid adverse effects.

Age (years), mean (± SD)	74.5 (±7.7)
Sex, *n* (%)	
Female	106 (76.8)
Male	32 (23.2)
BMI (kg/m^2^), mean (± SD)	26.47 (±4.65)
GCA subtype, *n* (%)	
Extracranial GCA	8 (5.8)
Cranial GCA	69 (50.0)
GCA without PMR	77 (55.8)
GCA with PMR	61 (44.2)
Ocular involvement, *n* (%)	12 (8.7)
Laboratory parameters at GCA onset, median (25th–75th percentile)	
CRP (mg/L)	56.0 (19.0–98.5)
ESR (mm/h)	69 (50–98)
Fibrinogen (mg/dL)	663 (536–883)
Drug therapy, *n* (%)	
Antiplatelet therapy	73 (52.9)
Oral anticoagulation	17 (12.3)
ACE inhibitors	43 (31.2)
Beta blockers	57 (41.3)
Calcium channel blockers	12 (8.7)
Diuretics	24 (17.4)
Other antihypertensives	15 (10.9)
Insulin	4 (2.9)
Metformin	12 (8.7)
Statins	45 (32.6)
DMARD	18 (13.0)
Methotrexate	15 (10.9)
Azathioprine	3 (2.2)
Relapse, *n* (%)	22 (15.9)
Major relapse	5 (3.6)
Minor relapse	17 (12.3)
Potential previous glucocorticoid adverse effects, *n* (%)	
Arterial hypertension	97 (70.3)
Diabetes mellitus	28 (20.3)
Obesity	24 (17.4)
Hyperlipidemia	92 (66.7)
Hypercholesterolemia	85 (61.6)
Hypertriglyceridemia	41 (29.7)
CKD	129 (93.5)
CKD 1	0 (0.0)
CKD 2	69 (49.3)
CKD 3	57 (41.3)
CKD 3a	48 (34.9)
CKD 3b	9 (6.5)
CKD 4	4 (2.9)
CKD 5	0 (0.0)
Osteoporosis	71 (51.4)
Bone fracture	25 (18.1)
Cataract	44 (31.9)
Glaucoma	13 (9.4)
Hepatic steatosis	14 (10.1)
Hepatic cirrhosis	0 (0.0)
VTE	12 (8.7)
Depression	8 (5.8)
Dementia	4 (2.9)
Gastritis	28 (20.3)
Peptic ulcer	6 (4.3)
Esophagitis	18 (13.0)
Pancreatitis	6 (4.3)
Number of potential previous glucocorticoid adverse effects, median (25th-75th percentile)	5 (3–6)

ACE, angiotensin-converting enzyme; BMI, body mass index; CKD, chronic kidney disease; CRP, C-reactive protein; DMARD, disease-modifying anti-rheumatic drug; ESR, erythrocyte sedimentation rate; GCA, giant-cell arteritis; PMR, polymyalgia rheumatica; VTE, venous thromboembolism.

### Development of glucocorticoid adverse effects during follow-up

Mean follow-up (± SD) duration in the GCA cohort was 87.1 ± 21.7 months. Any potentially new glucocorticoid adverse effect occurred in 104 patients with GCA (75.4%). Median of potentially new glucocorticoid adverse effect was one with a 25th-75thpercentile range of 0–3. Among newly developed glucocorticoid adverse effects, CKD progression was the most prevalent, occurring in 29% of the patients, followed by bone fractures in 23.2% and by cataracts in 18.1% of the patients. Development of new-onset arterial hypertension (12.3%), dementia (12.3%) and hyperlipidemia (10.9%) were additional common glucocorticoid adverse events. Further details of newly developed glucocorticoid adverse events during the follow-up period are listed in Table 2.

**Table 2:** Development of glucocorticoid adverse effects during the follow-up period.

Potential new glucocorticoid adverse effects, *n* (%)	
Arterial hypertension	17 (12.3)
Diabetes mellitus	5 (3.6)
Obesity	4 (2.9)
Hyperlipidemia	15 (10.9)
Hypercholesterolemia	13 (9.4)
Hypertriglyceridemia	10 (7.2)
New CKD	6 (4.3)
CKD stage progression	40 (29.0)
CKD 1	3 (2.2)
CKD 2	69 (50.0)
CKD 3	56 (40.6)
CKD 3a	38 (27.5)
CKD 3b	18 (13.0)
CKD 4	6 (4.3)
CKD 5	1 (0.7)
Osteoporosis	10 (7.2)
Bone fracture	32 (23.2)
Cataract	25 (18.1)
Glaucoma	3 (2.2)
Hepatic steatosis	3 (2.2)
Hepatic cirrhosis	0 (0.0)
VTE	10 (7.2)
Depression	5 (3.6)
Dementia	17 (12.3)
Gastritis	7 (5.1)
Peptic ulcer	1 (0.7)
Esophagitis	6 (4.3)
Pancreatitis	5 (3.6)
Patients with any potentially new glucocorticoid adverse effect, *n* (%)	104 (75.4)
Number of potentially new glucocorticoid adverse effects, median (25th-75th percentile)	1 (0–3)

CKD, chronic kidney disease; VTE, venous thromboembolism.

### Associations between clinical, laboratory and imaging parameters with glucocorticoid adverse effects

Significant association was observed between the occurrence of any relapse and new-onset diabetes mellitus (*p* = 0.025). Furthermore, a significant association was found between the initial glucocorticoid pulse therapy and the development of new-onset dementia (*p* = 0.041). No further significant associations were observed between initial glucocorticoid pulse therapy, any relapse, PMR and the occurrence of any other new adverse effects (Table 3). In simple logistic regression analysis, the occurrence of any relapse was significant associated with new-onset diabetes mellitus during follow-up (OR 9.58 [95% CI 1.50–61.37], *p* = 0.017) and remained a statistically significant predictor in multiple logistic regression analysis (OR 9.23 [95% CI 1.33–64.05], *p* = 0.025). Conversely, although the association between initial glucocorticoid pulse therapy and development of new-onset dementia was statistically significant in simple logistic regression analysis (OR 4.08 [95% CI 1.09–15.25], *p* = 0.036), no statistical significance could be achieved in multiple logistic regression analysis (OR 1.63 [95% CI 0.30–8.76], *p* = 0.571).

**Table 3:** Associations of new glucocorticoid adverse effects with clinical parameters of GCA with exact *p*-values of chi-square test.

	Initial glucocorticoid pulse therapy	Any relapse	PMR
Arterial hypertension	0.320	0.294	0.206
Diabetes mellitus	0.658	**0.025**	0.383
Obesity	>0.999	0.487	>0.999
Hyperlipidemia	>0.999	>0.999	0.421
Hypercholesterolemia	0.320	0.691	0.774
Hypertriglyceridemia	>0.999	>0.999	>0.999
CKD	>0.999	0.590	0.694
CKD stage progression	0.349	0.189	0.349
Osteoporosis	0.686	0.179	>0.999
Bone fracture	0.609	0.782	0.689
Cataract	0.279	>0.999	0.664
Glaucoma	0.267	>0.999	>0.999
Hepatic steatosis	0.341	0.393	0.583
Hepatic cirrhosis	-	-	-
VTE	0.333	0.359	0.337
Depression	0.546	>0.999	0.655
Dementia	**0.041**	0.469	0.801
Gastritis	>0.999	>0.999	0.464
Peptic ulcer	0.341	>0.999	>0.999
Esophagitis	>0.999	0.226	0.070
Pancreatitis	>0.999	0.568	0.655

CKD, chronic kidney disease; PMR, polymyalgia rheumatica; VTE, venous thromboembolism. Bold values indicate statistical significance *p* < 0.05.

No associations were identified between PWV >10 m/s or IMT ≥0.9 mm and the development of any new glucocorticoid adverse effects. Additionally, no significant association was noted between ESR >30 mm/h or CRP >5 mg/L, neither at study inclusion nor at GCA onset, and the development of any new glucocorticoid adverse effects (Table 4). In correlation analysis, no significant correlations were found between the number of newly developed glucocorticoid adverse effects and imaging or laboratory parameters of endothelial dysfunction and inflammation at study inclusion (Table 5). Significant correlations were found between the number of newly developed glucocorticoid adverse effects and CRP at GCA onset (*r* = 0.297, *p* = 0.006) and fibrinogen at GCA onset (*r* = 0.351, *p* = 0.002), but not for ESR at GCA onset (*r* = 0.105, *p* = 0.387).

**Table 4:** Associations of potential new glucocorticoid adverse effects with imaging and laboratory parameters at study inclusion and from GCA onset with exact *p*-values of chi-square test.

	PWV > 10 m/s	IMT ≥ 0.9 mm	ESR > 30 mm/h at study inclusion	CRP > 5 mg/L at study inclusion	ESR > 30 mm/h at GCA onset	CRP > 5 mg/L at GCA onset
Arterial hypertension	0.781	0.448	0.642	0.597	0.999	>0.999
Diabetes mellitus	0.149	0.655	0.369	0.648	0.384	>0.999
Obesity	0.274	0.629	0.307	0.645	>0.999	>0.999
Hyperlipidemia	>0.999	0.144	0.348	0.270	>0.999	>0.999
Hypercholesterolemia	0.761	0.345	0.276	0.766	>0.999	>0.999
Hypertriglyceridemia	>0.999	0.184	>0.999	>0.999	>0.998	>0.999
CKD	0.216	0.405	>0.999	>0.999	>0.999	0.729
CKD stage progression	>0.999	0.562	>0.999	>0.999	0.609	0.427
Osteoporosis	0.519	0.750	0.176	0.740	>0.999	>0.999
Bone fracture	0.664	0.138	0.272	0.213	0.516	0.595
Cataract	0.350	0.649	0.403	0.653	0.626	0.517
Glaucoma	>0.999	0.194	>0.999	0.279	>0.999	>0.999
Hepatic steatosis	>0.999	0.254	>0.999	>0.999	>0.999	>0.999
Hepatic cirrhosis	–	–	–	–	–	–
VTE	0.519	0.508	0.570	0.513	0.998	0.593
Depression	>0.999	>0.999	>0.999	0.156	>0.999	>0.999
Dementia	0.563	>0.999	0.359	>0.999	>0.999	0.826
Gastritis	>0.999	>0.999	>0.999	0.381	>0.999	0.357
Peptic ulcer	0.400	>0.999	>0.999	0.393	>0.999	>0.999
Esophagitis	>0.999	>0.999	>0.999	>0.999	>0.999	>0.999
Pancreatitis	0.649	>0.999	>0.999	0.156	>0.999	>0.999

CKD, chronic kidney disease; CRP, C-reactive protein; ESR, erythrocyte sedimentation rate; IMT, intima-media-thickness; PWV, pulse-wave velocity; VTE, venous thromboembolism.

**Table 5:** Correlations of imaging and laboratory parameters at study inclusion with number of newly developed glucocorticoid adverse effects.

	Number of newly developed glucocorticoid adverse effects	
	*r*	*p*-value
PWV	0.018	0.848
Aix	0.079	0.389
Carotid IMT	−0.105	0.231
Femoral IMT	−0.015	0.828
Subclavian IMT	0.112	0.093
ADMA	0.029	0.739
SDMA	−0.082	0.354
CRP	0.042	0.628
ESR	−0.014	0.879
Fibrinogen	0.076	0.556
WBC	−0.083	0.335
Neutrophils	0.032	0.711
Monocytes	−0.134	0.120
Lymphocytes	−0.090	0.294
CD subtypes		
CD3 cells	−0.063	0.464
CD3 + CD4+ cells	−0.030	0.728
CD3 + CD8+ cells	−0.072	0.405
CD4/CD8 ratio	0.077	0.373
CD3-CD16 + CD56+ cells	−0.119	0.167
CD19 cells	−0.025	0.776
CD45 cells	−0.074	0.392
CD4+/CD28-cells	0.015	0.867
CD8+/CD28-cells	0.044	0.617

ADMA, asymmetric dimethylarginine; Aix, augmentation index; CRP, C-reactive protein; ESR, erythrocyte sedimentation rate; IMT, intima-media-thickness; PWV, pulse-wave velocity; SDMA, symmetric dimethylarginine; WBC, white blood cells.

## Discussion

By our retrospective analysis of GCA patients, we demonstrated a high number of potential glucocorticoid adverse effects which was comparable to previous studies. Proven et al. (10) described glucocorticoid adverse effects in 86% of patients with GCA over a median follow-up period of ten years while we observed any new glucocorticoid adverse effect in 75% of patients with GCA over a mean follow-up period of 7.25 years. Regarding the total amount of newly developed glucocorticoid adverse effects, our study was also comparable to another previous study by Perrineau et al. (11), who reported the same median adverse effect event number but lower 25th-75th percentiles ranging from 0–1 adverse effects. In our study, 25th-75th percentiles ranged from 0–3 adverse effects, while the follow-up period was larger than by Perrineau et al. (11) (78.1 vs. 34 months). This may be also an explanation for the higher percentile range in our study. Other demographics, like gender or age which may influence and contribute to diseases as defined in our study as glucocorticoid adverse effects, were also comparable to previous studies (10, 11). Nevertheless, we observed some changes of each specific glucocorticoid adverse effects compared to previous studies. The most common glucocorticoid adverse effect in our analysis was CKD stage progression, occurring in 29% of the cases, although a clear reason for the high rate of CKD progression remains elusive due to the retrospective study design. One potential and probably the main cause for CKD progression was the aging process of the patient cohort during the observational period, as 93.5% of our GCA patients had CKD grade 2–5 at study inclusion. Other causes may be inadequate treatment of concomitant arterial hypertension or diabetes mellitus and also the intake of potential other nephrotoxic drugs during the observational period. Nevertheless, potential direct nephrotoxic effect of glucocorticoid therapy, but also indirect nephrotoxic effects of glucocorticoid therapy due to worsening of concomitant arterial hypertension or diabetes mellitus cannot be excluded. However, the overall rate of clinical relevant CKD was low in our cohort as only six patients and one other patient had CKD grade 4 and grade 5, respectively, at the end of the observational period. Nevertheless, the high rate of CKD progression sets our analysis apart from existing literature, where cataract and bone fracture were identified as the most prevalent adverse effect of glucocorticoid therapy (10, 11, 24). We recorded bone fracture in 23.2% of the patients as the second and cataract in 18.1% of the patients as the third most common adverse effect, followed by arterial hypertension and dementia which were recorded each in 12.3% of cases. Most rates of the respective glucocorticoid adverse effects were lower compared to Proven et al. (10) (38, 41, 22%, not recorded, respectively), but were higher to Perrineau et al. (11) (13, 8, 8%, not recorded, respectively). Regarding dementia, another study reported only a rate of 0.6% of GCA patients which is twentyfold lower than in our study (25). New-onset hyperlipidaemia were two-fold higher than in the cohort from Perrineau et al. (11) while new-onset diabetes mellitus were lower than in the cohort from Proven et al. (10). Rates of other new-onset specific glucocorticoid adverse effects, including VTE or gastrointestinal disorders, were not reported by both studies. Compared to other studies, however, we observed in our GCA cohort higher rates for VTE and gastritis with lower rates of glaucoma and peptic ulcers (25–27). To the best of our knowledge, no previously reported incidence rates for hepatic steatosis and cirrhosis, depression, esophagitis and pancreatitis in GCA patients were found.

The high rate of glucocorticoid adverse effects observed in our GCA cohorts can be explained on the one hand by the necessitated high doses, particularly in cases of ocular involvement and of GCA relapse, and on the other hand by the substantial proportion of older patients, which generally increases the likelihood for numerous diseases, including those which were defined in this study as glucocorticoid adverse effects. Causes for the different incidence rates of glucocorticoid adverse effects between our analysis and previous studies are various. Firstly, different rates of glucocorticoid adverse effects may be attributed to a stricter prevention regime for several glucocorticoid adverse effects, including calcium supplementation, administration of proton pump inhibitors or antihypertensive drugs. Especially, older studies on this topic, when knowledge of potential glucocorticoid adverse effects and their prevention was sparse, may report higher adverse effect rates than newer studies. Also the increasing use of disease-modifying anti-rheumatic drugs in GCA with their glucocorticoid sparing effect may cause a decrease of glucocorticoid adverse effects (28). Another aspect may be the missing awareness of less typical glucocorticoid adverse effects like VTE or glaucoma, which have been reported only occasionally for other diseases or in newer studies. Furthermore, some glucocorticoid adverse effects have not been investigated yet in GCA, like pancreatitis or a worsening of renal insufficiency. It must be, however, noted that especially CKD stage progression but also gastritis or hepatic steatosis may be caused also by several other factors including aging, smoking, or secondary to other drugs and other diseases. Lastly, the observed rates of some glucocorticoid adverse effects may differ due to the fact that not every incidence of the respective glucocorticoid adverse effect may be reported by the MEDOCS system. In case of slight gastritis, arterial hypertension or asymptomatic hepatic steatosis, which can be managed by resident physicians without necessary hospitalization, those incidences were not reported in MEDOCS.

Predictors for adverse effects of glucocorticoid therapy in GCA have been rarely investigated to the best of our knowledge. In the study from Perrineau et al. (11), age > 75 years, occurrence of relapse and a past medical history of diabetes were significant predictors for glucocorticoid adverse effects. However, the predictive role of other clinical, imaging and laboratory parameters remains elusive. In our study, we observed statistically significant associations between the occurrence of any relapse and the new-onset diabetes mellitus as well as between initial glucocorticoid pulse therapy and new-onset dementia. While both associations were significant in simple regression analysis, new-onset dementia failed to be statistically significant in multiple regression analysis. This may be explained by the fact that, with the occurrence of any relapse, glucocorticoid dosages typically increase and raising thereby the risk for the development of new-onset diabetes mellitus. Regarding the new onset of dementia, a systematic review has revealed that in the majority of studies examining all-cause dementia or Alzheimer’s disease in relation to glucocorticoid use, there is either no association or a negative associations suggesting even potential protective effects for glucocorticoids (29). However, vascular dementia was commonly excluded and this fact may be an explanation for these contradictory data as another study reported that the risk of vascular dementia is increased under the use of glucocorticoids and nonsteroidal anti-inflammatory drugs (13). Due to the lack of differentiation in dementia subtypes in our study, we are unable to determine which dementia subtypes have developed in GCA patients. Additionally, potential influence by other cardiovascular risk factors for the development of new-onset dementia can be assumed, especially as multiple regression analysis including cardiovascular variables did not revealed statistical significance. Prevalence of concomitant arterial hypertension, diabetes mellitus and hyperlipidemia at study inclusion was high in our GCA cohort, but similar or only slightly divergent compared to other GCA cohorts (30, 31). Thus, also the risk for cardiovascular events and ischemic complications like stroke may be increased which may lead ultimately to higher rates of new-onset dementia (32).

Interestingly, no further associations between clinical, imaging and laboratory parameters were found, especially on those parameters which may be influenced by glucocorticoid administration like inflammatory parameters or parameters of endothelial dysfunction, except for CRP and fibrinogen at GCA onset and the number of newly developed glucocorticoid adverse effects. However, due to the retrospective study design with missing systematic screening, we cannot reliably differentiate between the time from GCA onset to study inclusion if one of our defined potential glucocorticoid adverse effect was a genuine adverse effect or rather a comorbidity. Due to that insufficient discrimination, the presented significant correlations of CRP and fibrinogen at GCA onset reflect only the results on the number of newly developed glucocorticoid adverse effects after study inclusion. Additionally, as the same inflammatory parameters at study inclusion did not correlated with the number of newly developed glucocorticoid adverse effects anymore, the predictive role of CRP and fibrinogen on glucocorticoid adverse effect seem to be negligible. Glucocorticoid administration typically goes along with a reduction in inflammatory parameters. In a recent cohort study from Japan, however, 30% of patients with PMR still exhibited elevated values of inflammatory parameters above the norm after 52 weeks of therapy. The cumulative incidence of glucocorticoid dosage increase associated with elevated CRP levels was 34.9% over the 52-week follow-up period. Therefore, initially, we expected an association between inflammatory markers and the emergence of glucocorticoid adverse effects (33). Similarly, other studies have demonstrated that aortic PWV decreases and aortic PWV is correlated with the percentage change in plasma CRP in patients with GCA and PMR under glucocorticoid therapy (34, 35). Hafner et al. (36) reported that glucocorticoid administration in patients with GCA had been associated with a reduction of carotid IMT. The expectation that, conversely, increased values of PWV and IMT were associated with potential new-onset of glucocorticoid adverse effects could not be therefore confirmed. Other parameters of inflammation or endothelial dysfunction, including lymphocyte subsets, ADMA, SDMA or Aix, did also not correlate with number of newly developed glucocorticoid adverse effect assuming that other pathways than inflammation and endothelial dysfunction may contribute to adverse effects of glucocorticoids.

Limitations of this study are the retrospective study design, absent control group and the missing systematic screening for all respective glucocorticoid adverse effects at study inclusion and during follow-up. Especially, a sufficient discrimination if one of our defined potential glucocorticoid adverse effect was a genuine adverse effect or an undocumented comorbidity between the time of GCA onset and study inclusion cannot be made by this sub-study design. As mentioned above, potentially developed glucocorticoid adverse effects, which have been diagnosed and treated at a resident physician, were not documented in MEDOCS and may be missed by our chart review. Additionally, glucocorticoid adverse effects which may occurred prior to study inclusion but were documented by MEDOCS at a later stage may be unintentionally attributed as newly developed adverse effects. Furthermore, many glucocorticoid adverse effects are dose-dependent while this analysis did not evaluate the exact glucocorticoid dosage (37). In addition, cumulative dose of glucocorticoids could not be reliably recorded and a reliable discrimination between an underlying comorbidity prior to GCA diagnosis and a genuine potential previously known glucocorticoid adverse effect cannot be made due to the retrospective sub-study design. Thus, no associations about the cumulative glucocorticoid dose could be made although glucocorticoid adverse effects seem to be dose and time dependent (9–11). Moreover, potential bias by other concomitant drugs, like osteoporosis prophylaxis or disease-modifying anti-rheumatic drugs, or by other diseases which may influence the incidence of glucocorticoid adverse effects in this study needs to be mentioned, while exact therapy durations or dosages of concomitant drugs could not reliably recorded due to the retrospective sub-study design.

In conclusion, our study demonstrated high incidence rates of glucocorticoid adverse effects over a long-term observational period and suggesting that relapse of GCA may be a clinical predictor for the development of diabetes mellitus in GCA patients. Laboratory and imaging parameters are not suitable predictors for glucocorticoid adverse effects. Prospective studies with close monitoring and dosage documentation and clinical trials investigating further alternative treatment modalities are needed for a comprehensive understanding of the risk–benefit profile of glucocorticoid therapy and to mitigate the burden of glucocorticoid adverse effects while maintaining therapeutic efficacy in patients with GCA.

## Data availability statement

The original contributions presented in the study are included in the article/supplementary material, further inquiries can be directed to the corresponding author.

## Ethics statement

The studies involving humans were approved by ethics committee of the medical university of Graz. The studies were conducted in accordance with the local legislation and institutional requirements. The participants provided their written informed consent to participate in this study. Written informed consent was obtained from the individual(s) for the publication of any potentially identifiable images or data included in this article.

## Author contributions

LS: Formal analysis, Validation, Visualization, Writing – original draft, Writing – review & editing. FH: Conceptualization, Methodology, Project administration, Supervision, Writing – review & editing. AM: Formal analysis, Investigation, Resources, Writing – review & editing. MB: Resources, Supervision, Writing – review & editing. CD: Formal analysis, Investigation, Writing – review & editing. PJ: Data curation, Formal analysis, Writing – review & editing.
